# Kynurenine and Hemoglobin as Sex-Specific Variables in COVID-19 Patients: A Machine Learning and Genetic Algorithms Approach

**DOI:** 10.3390/diagnostics11122197

**Published:** 2021-11-25

**Authors:** Jose M. Celaya-Padilla, Karen E. Villagrana-Bañuelos, Juan José Oropeza-Valdez, Joel Monárrez-Espino, Julio E. Castañeda-Delgado, Ana Sofía Herrera-Van Oostdam, Julio César Fernández-Ruiz, Fátima Ochoa-González, Juan Carlos Borrego, Jose Antonio Enciso-Moreno, Jesús Adrián López, Yamilé López-Hernández, Carlos E. Galván-Tejada

**Affiliations:** 1 Unidad Académica de Ingeniería Eléctrica, Universidad Autónoma de Zacatecas, Jardín Juárez 147, Centro, Zacatecas 98000, Mexico; jose.celaya@uaz.edu.mx (J.M.C.-P.); kvillagrana@uaz.edu.mx (K.E.V.-B.); 2 Unidad de Investigación Biomédica de Zacatecas, Instituto Mexicano del Seguro Social, Centro, Zacatecas 98000, Mexico; intrinsection@hotmail.com (J.J.O.-V.); jecastanedade@conacyt.mx (J.E.C.-D.); julio137.jc@gmail.com (J.C.F.-R.); fatis_8a@icloud.com (F.O.-G.); enciso_2000@yahoo.com (J.A.E.-M.); 3 Department of Health Research, Christus Muguerza del Parque Hospital Chihuahua, University of Monterrey, San Pedro Garza García 66238, Mexico; joel.monarrez@udem.edu; 4 Cátedras-CONACyT, Consejo Nacional de Ciencia y Tecnología, Ciudad de México 03940, Mexico; 5 Doctorado en Ciencias Biomédicas Básicas, Centro de Investigación en Ciencias de la Salud y Biomedicina, Universidad Autónoma de San Luis Potosí, San Luis Potosí 78210, Mexico; sofiaherreravo@alumnos.uaslp.edu.mx; 6 Área de Ciencias de la Salud, Universidad Autónoma de Zacatecas, Carretera Zacatecas–Guadalajara kilometro 6, Ejido la Escondida, Zacatecas 98160, Mexico; 7 Departamento de Epidemiología, Hospital General de Zona #1 “Emilio Varela Luján”, Instituto Mexicano del Seguro Social, Centro, Zacatecas 98000, Mexico; juan.borrego@imss.gob.mx; 8 Laboratorio de MicroRNAs y Cáncer, Unidad Académica de Ciencias Biológicas, Universidad Autónoma de Zacatecas, Zacatecas 98000, Mexico; jalopez@uaz.edu.mx; 9 Metabolomics and Proteomics Laboratory, Autonomous University of Zacatecas, Zacatecas 98000, Mexico

**Keywords:** COVID-19, sex, machine learning, metabolomics

## Abstract

Differences in clinical manifestations, immune response, metabolic alterations, and outcomes (including disease severity and mortality) between men and women with COVID-19 have been reported since the pandemic outbreak, making it necessary to implement sex-specific biomarkers for disease diagnosis and treatment. This study aimed to identify sex-associated differences in COVID-19 patients by means of a genetic algorithm (GALGO) and machine learning, employing support vector machine (SVM) and logistic regression (LR) for the data analysis. Both algorithms identified kynurenine and hemoglobin as the most important variables to distinguish between men and women with COVID-19. LR and SVM identified C10:1, cough, and lysoPC a 14:0 to discriminate between men with COVID-19 from men without, with LR being the best model. In the case of women with COVID-19 vs. women without, SVM had a higher performance, and both models identified a higher number of variables, including 10:2, lysoPC a C26:0, lysoPC a C28:0, alpha-ketoglutaric acid, lactic acid, cough, fever, anosmia, and dysgeusia. Our results demonstrate that differences in sexes have implications in the diagnosis and outcome of the disease. Further, genetic and machine learning algorithms are useful tools to predict sex-associated differences in COVID-19.

## 1. Introduction

Sex differences in manifestations of viral infections have been observed for multiple respiratory viruses [1,2] where men have shown higher disease severity and mortality compared with women, including SARS-CoV [3], MERS-CoV [4], the H1N1 pandemic [5], and others. A recent meta-analysis of 3.1 million global cases showed that men have a nearly three times higher chance of being admitted to an intensive care unit (ICU) and a higher risk of dying, even though the incidence of COVID-19 infection is similar [6]. In addition, laboratory measures of routinely collected blood and urine samples from infected individuals have revealed differential patterns by sex and age [7]. Researchers have also looked at sex to understand the mechanisms behind the differences in COVID-19 outcome [8], with some studies focusing on the role of hormones, adipose tissue distribution, and metabolites [9,10,11,12].

In recent years, machine learning (ML) has been widely used for biomarker discovery [13,14,15]. Support vector machine (SVM), firstly proposed by Vapnik [16] has proved to be a powerful technique for pattern recognition, classification, and regression in many fields [17,18,19,20]. SVMs are supervised learners to construct models from available training data with a known classification. To obtain accurate class predictions, SVMs provide a number of free parameters that have to be tuned to reflect the requirements of a given task. Logistic regression (LR) is another technique borrowed by ML from the field of statistics [21]. Genetic algorithms (GA) are ML search techniques inspired by Darwinian evolutionary models. GA are metaheuristics that imitate the long-term optimization process of biological evolution for solving mathematical optimization problems [22].

ML has already been used to build survival and prognostic prediction models in cancer [15,23,24], Alzheimer’s disease [25], and obstructive sleep apnea [26]. Similarly, efforts to develop novel diagnostic approaches for COVID-19 using ML algorithms have been proposed [27]. Despite not being focused specifically on sex differences, recent works have reported the use of artificial intelligence (AI) to predict COVID-19 outcomes using clinical data. Jiang et al. [28] used traditional ML methods such as decision tree (DT), random forest (RF), and SVM to predict disease progression to acute respiratory distress syndrome (ARDS) in COVID-19 patients with a 70%–80% overall accuracy. In another study, Xu et al. [29] tested five algorithms for modeling, including LR, RF, SVM, DT, and deep neural networks (DNN) leading to the identification of 19 risk factors to determine whether the patient would develop ARDS: severity evaluation at admission, sex, age, body mass index (BMI), temperature, cough, shortness of breath, hemoptysis, hypertension, diabetes, secondary bacterial infection, lung consolidation, lymphocyte count, CK, NLR, ALT, AST, LDH, and CRP. However, there are still limited data on sex differentials in COVID-19 outcomes.

Jiang et al., algorithmically identified the combinations of clinical characteristics of COVID-19 that predict outcomes, developing a tool with AI capabilities that predicted patients at risk for more severe illness on initial presentation. A mildly elevated alanine aminotransferase (ALT), the presence of myalgias, and an elevated hemoglobin were the clinical features, on presentation, that were the most predictive. The predictive models that learned from historical data of patients from the studied population achieved high accuracy levels in predicting severe cases [28]. Lu et al., employed a neural network algorithm to predict ICU admission, finding that C-reactive protein, lactate dehydrogenase, creatinine, white-blood cell count, D-dimer and lymphocyte count, showed temporal divergence between COVID-19 patients hospitalized in the general floor who were upgraded to ICU compared to those who were not [30]. Similarly, Li et al. developed a deep neural network model and a risk-score system to predict ICU admission and in-hospital mortality. Prediction performance used the receiver operating characteristic area under the curve (AUC). In this study, the authors found that procalcitonin, lactate dehydrogenase, C-reactive protein, ferritin, and oxygen saturation were the top ICU predictors, while the top mortality predictors were age, lactate dehydrogenase, procalcitonin, cardiac troponin, C-reactive protein, and oxygen saturation [31]. By using machine learning, Hou et al. identified age, procalcitonin, C-creative protein, lactate dehydrogenase, D-dimer, and lymphocytes as the top mortality predictors. The top six ICU admission predictors were procalcitonin, lactate dehydrogenase, C-creative protein, pulse oxygen saturation, temperature, and ferritin [32].

Ancochea et al. [33] identified sex-dependent differences in clinical features, diagnosis, treatment, and hospital resource use associated with COVID-19 using Natural Language Processing and ML.

Understanding how sex could influence COVID-19 outcomes can have relevant implications for accurate clinical management and the implementation of mitigation strategies. The rapid development of automated diagnostic systems based on artificial intelligence and ML can thus contribute to increasing the speed of patient profiling to help improve the management of the COVID-19 pandemic.

## 2. Materials and Methods

### 2.1. Patients, Sample Collection, and Processing

#### 2.1.1. Patients Enrollment and Sample Collection

Clinical data from a sample of 157 patients were extracted from the epidemiologic data set of patients admitted to the Respiratory Triage at the General Hospital of the Mexican Institute of Social Security from March to November 2020 in the city of Zacatecas. Plasma samples from these patients were collected at an early stage of the disease (four days on average after onset of symptomatology and prior to diagnosis). Forty individuals suspected of infection due to close contact with a COVID-19 case tested negative (18 men, 22 women) and 117 (68 men, 49 women) had a positive result using reverse transcriptase polymerase chain reaction (RT-qPCR); in these patients, plasma samples were collected within two days of hospitalization, prior to antibiotic use, if any. A description of the clinical features including demographic data, clinical symptoms, and laboratory variables is provided in Table 1. The study protocol was written in accordance with the Declaration of Helsinki and approved by the Ethics Committee of the Mexican Institute for Social Security (ID: R-2020-785-068).

#### 2.1.2. Metabolomics Profile of Plasma Samples

Metabolites were measured using a locally developed LC-MS/MS metabolomics assay previously developed for urine and adapted to work with plasma [34]. Mass spectrometric analyses were performed on an ABSciex 4000 Qtrap tandem MS instrument (Applied Biosystems/MDS Analytical Technologies, Foster City, CA, USA) equipped with an Agilent 1260 series UHPLC system (Agilent Technologies, Palo Alto, CA, USA). The method combines the derivatization and extraction of the analytes and the selective mass-spectrometric detection using multiple reaction monitoring (MRM) pairs.

Amino acids, biogenic amines and derivatives, and organic acids were analyzed by a reverse-phase LC-MS/MS custom assay, while glycerophospholipids, acylcarnitines, glucose, and sphingomyelins were measured by direct injection (DI).

#### 2.1.3. Sample Preparation

A working internal standard (ISTD) solution mixture in water (for amino acids, biogenic amines, carbohydrates, carnitines and derivatives, and phosphatidylcholines and their derivatives) was made by mixing all the prepared isotope-labeled stock solutions together. For organic acids, a working internal standard (ISTD) solution mixture in aqueous methanol was made. All standard solutions were aliquoted and stored at −80 °C until further use. 2H-, 13C-, and 15N-labeled compounds were purchased from Cambridge Isotope Laboratories, Inc. (Tewksbury, MA, USA) and from Sigma-Aldrich (Oakville, ON, Canada). All other standards including lactic acid, beta-hydroxybutyric acid, alpha-ketoglutaric acid, citric acid, butyric acid, isobutyric acid, propionic acid, p-hydroxyhippuric acid, succinic acid, fumaric acid, pyruvic acid, hippuric acid, methylmalonic acid, homovanillic acid, indole-3-acetic acid, uric acid, and their isotope-labeled standards were all purchased from Sigma-Aldrich (Oakville, ON, Canada).

For organic acid analysis, 150 µL of ice-cold methanol and 10 µL of isotope-labeled internal standard mixture [34] were added to 50 µL of plasma sample for overnight protein precipitation at −20 °C, followed by centrifugation at 13,000× *g* for 20 min. A total of 50 µL of supernatant was loaded into the center of wells of a 96-deep-well plate, followed by the addition of 3-nitrophenylhydrazine reagent. Butylated hydroxytoluene stabilizer (2 mg/mL) and water were added before LC-MS injection.

For amino acids and biogenic amines and derivatives, glycerophospholipids, acylcarnitines, and sphingomyelins, samples were thawed on ice and subsequently vortexed and centrifuged at 13,000× *g*; 10 μL of each sample was then loaded onto the center of the filter on the upper 96-well plate and dried in a stream of nitrogen. Subsequently, phenyl-isothiocyanate was added for derivatization. After incubation, the filter spots were dried again using an evaporator. Extraction of the metabolites was then achieved by adding 300 μL of extraction solvent.

#### 2.1.4. LC-MS/MS Method

An Agilent reversed phase Zorbax Eclipse XDB C18 column (3.0 mm × 100 mm, 3.5 μm particle size, 80 Å pore size) with a Phenomenex (Torrance, CA, USA) SecurityGuard C18 pre-column (4.0 mm × 3.0 mm) were used. The LC parameters used were as follows: mobile phase A was 0.2% (v/v) formic acid in water, and mobile phase B was 0.2% (v/v) formic acid in acetonitrile. The gradient profile was as follows: t = 0 min, 0% B; t = 0.5 min, 0% B; t = 5.5 min, 95% B; t = 6.5 min, 95% B; t = 7.0 min, 0% B; and t = 9.5 min, 0% B. The column oven was set at 50 °C. The flow rate was 500 μL/min, and the sample injection volume was 10 μL.

For the analysis of organic acids, the mobile phases used were (A) 0.01% (v/v) formic acid in water, and (B) 0.01% (v/v) formic acid in methanol. The gradient profile was as follows: t = 0 min, 30% B; t = 2.0 min, 50% B; t = 12.5 min, 95% B; t = 12.51 min, 100% B; t = 13.5 min, 100% B; t = 13.6 min, 30% B; and finally maintained at 30% B for 4.4 min. The column oven was set to 40 °C. The flow rate was 300 μL/min, and the sample injection volume was 10 μL.

#### 2.1.5. DI-MS/MS Method

The LC autosampler was connected directly to the MS ion source by red PEEK tubing. The mobile phase was prepared by mixing 60 μL of formic acid, 10 mL of water, and 290 mL of methanol; and the flow rate was programmed as follows: t = 0 min, 30 μL/min; t = 1.6 min, 30 μL/min; t = 2.4 min; 200 μL/min; t = 2.8 min, 200 μL/min; and t = 3.0 min, 30 μL/min. The sample injection volume was 20 μL.

#### 2.1.6. Quantification

To quantify organic acids, amino acids, biogenic amines, and derivatives, an individual seven-point calibration curve was generated for each analyte. The ratios of each analyte’s signal intensity to its corresponding isotope-labeled internal standard were plotted against the specific known concentrations using quadratic regression with a 1/x^2^ weighting.

Lipids, acylcarnitines, and glucose were analyzed semiquantitatively. Single point calibration of a representative analyte was built, using the same group of compounds that share the same core structure, assuming linear regression through zero. All data analysis was conducted using Analyst 1.6.2 and MultiQuant 3.0.3. Metabolites with more than 50% of missing values were removed from further analysis.

#### 2.1.7. Cytokine and Chemokine Quantification in Plasma Samples

We used a bead based flow cytometry assay (Legendplex, Biolegend, San Diego, CA, USA) for the quantitative simultaneous determination of 13 analytes: IL-1β, IFN-α, IFN-γ, TNF-α, IP-10, IL-6, IL-8 (CXCL8), IL-10, IL-12p70, GM-CSF, IFN-β, and IFN-λ. The assays were performed according to the manufacturer protocols and procedures. Pre-coated beads were dispensed in a 96 well filter plate and mixed with either the plasma samples or standards. Detection was made by biotin labeled antibodies and PE-Streptavidin detection reagents. The flow cytometry data were acquired in a FACS CANTO II flow cytometer (BD Biosciences, Franklin Lakes, NJ, USA) and analyzed in the FirePlex software (Biolegend, USA). Regression analysis was calculated, and the limit of detection and limit of quantification was obtained for each molecule (R^2^ value > 0.995).

### 2.2. Statistical Analysis

#### 2.2.1. Descriptive Statistics

Medians (interquartile ranges [IQRs]) and frequency (%) were used to report healthy controls and patient baseline characteristics for continuous and categorical variables, respectively. This information is shown in Table 1. Normality was assessed by the D’Agostino-Pearson normality test. Continuous variables were compared using Mann–Whitney U tests or Kruskal–Wallis tests, and categorical variables (sex, smoking, symptoms, and comorbidities) were compared using the chi-square test for trend, with p values of less than 0.05 considered statistically significant. The analyses were conducted using GraphPad Prism version 8.0.1 for Windows (GraphPad Software, La Jolla, CA, USA).

#### 2.2.2. Machine Learning Methodology

**Figure 1:**
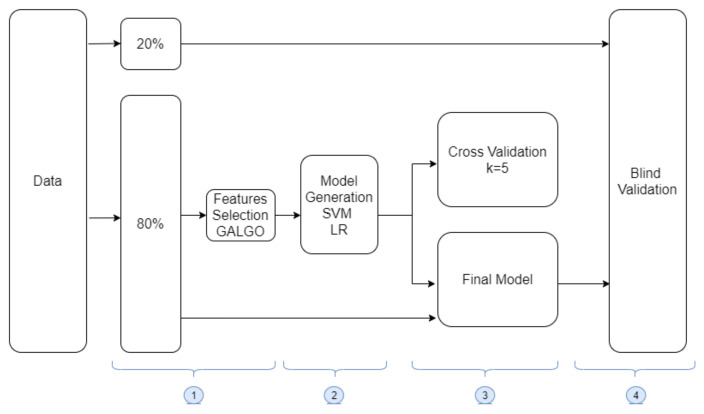
Flow chart representing the process of features selection, machine learning models generation (by Logistic Regression and Support Vector Machine), and validation (cross-validation and blind validation).

To assess clinical, immunologic, and metabolic associations with sex, a genetic approach was used to identify features that could be used in multivariate modeling to predict COVID-19 by sex. The proposed methodology is presented in Figure 1. It consists of four stages: (1). Data are split into training/testing; a blind test is used to search and find the best features to predict COVID-19 using a genetic algorithm; (2). Representative models are created using SVM and LR; (3). Model training and cross-validation are performed; and (4). Final models of SVM and LR are tested on an unseen blind data set to establish the model robustness on new samples.

#### 2.2.3. Data Preparation and Feature Selection

Four patients were excluded from the ML techniques due to several missing variables, but imputation with the mean was performed for patients with an individual missing variable; the mean was calculated with respect to the subgroup to which each patient belonged, that is, whether they were male or female, control patient or outpatient, hospitalized or in critical condition due to COVID-19. The age and binary variables (0, 1) were excluded for data normalization, which consisted of the conversion of values to z-scores representing standard deviations below or above the mean of a reference population, Equation (1), where x = the observed measure, μ is population mean, and σ is the population standard deviation [35].

(1) z=(x−μ)/σ

Figure 1, stage 1, shows the selection of variables using a genetic algorithm (GALGO, R package) designed to develop multivariate statistical models [36]. The parameters included were goal Fitness = 1, maxBigBangs = 1000, and maxGenerations = 300; these were used with two classification methods, LR and SVM. One thousand models were generated to obtain a fitness goal closer to 1. With each model, the selected features produced a ranking graphic from the most to the less frequent to ultimately build the optimal model.

The first ML technique used was LR, a transformed version of linear regression using the logic function, which was useful to model the probability of an event given other variables, namely, the probability of belonging to a group based on predicted probabilities from 0 to 1, which is considered the standard classification method for binary problems [37]. The model inputs real values that are multiplied by a weight and the sum is entered to the logit function Equation (2), to obtain the probability of belonging to one or another group based on the function of the threshold value [38,39,40], where z is the linear sum α plus $\beta_{1}$ by $X_{1}$ plus $\beta_{2}$ by $X_{2}$, and so on up to $\beta_{k}$ times $X_{k}$, where the Xs are the independent variables of interest, α the constant term, and $\beta_{i}$ (slopes) representing the unknown parameters.

(2) $$z=\alpha +\beta_{1}X_{1}+\beta_{2}X2+...\beta_{k}X_{k}$$

The second ML technique was SVM, a group of algorithms used for classification and regression. It is a model that represents sample points in space, splitting two classes of a new sample by means of a separation hyperplane, defined as the vector between the two points of the two classes, closer to what is called the support vector [41]. Using the simple mathematical Equation (3), it allows linear division of the domain [41]. Here, y is the optimal hyperplane, and γ is the constant that indicates the position of the hyperplane with respect to the origin of coordinates. This constant is called the bias, and w is the normal vector of the hyperplane.

(3) y=wx+γ

Ten experiments were performed, half using LR and the other half SVM, to predict patients without COVID-19 versus infected patients. First, all patients were included regardless of sex to identify all relevant characteristics associated with COVID-19 infection, followed by a second experiment with only women, and a third one with only men. The fourth and fifth experiments aimed at predicting sex among infected and non-infected patients, respectively.

#### 2.2.4. Model Generation

Once the variables were selected by GALGO in the experiments, wrapping techniques were implemented. Forward selection (FS) was used, this is an iterative method that starts without variables in the model, and with each iteration variables are added one by one; if the performance of the model improves, then variables are added until no improvement in the classification model is achieved [42]. For each experiment, the possible models were presented, and the one that produced the highest level of prediction was chosen as the biomarker. This model is therefore capable of optimizing the prediction of the patient group. Two models were obtained for each of the experiments, corresponding to the LR and SVM models. This stage was carried out within stage 2 (Figure 1).

#### 2.2.5. Model Training and Validation

Once the best model was chosen, it was used with 80% of the data set, and k-fold cross-validation was also performed (k = 5 from Figure 1 stage 4), a useful technique to evaluate the effectiveness of the model that mitigates overfitting, in which one of the subsets is used as test data and the rest as training data. Finally, the average of the results of each iteration is computed to obtain a single result and the performance of the proposed model.

#### 2.2.6. Blind Testing

The model was then subjected to evaluation or testing with the 20% of the remaining data, namely, with data unknown to the model resembling an evaluation in an unknown population, for instance, from another state. This is shown in stage 4 of Figure 1.

#### 2.2.7. Models Evaluation Metrics

For cross-validation, training, and blind testing, models were evaluated using the following metrics:

Sensitivity: The ability of the test to detect the infection in infected individuals, calculated with the ratio of true positives (TP), divided by the sum of false negatives (FN) and true positives (TP), (Equation (4)).

(4) Sensitivity=TP/FN+TP

Specificity: The ability of the test to detect negative cases among the healthy, calculated by dividing the true negatives (TN) by the sum of the false positives (FN) and true negatives (TN), (Equation (5)).

(5) Specificity=TN/FP+TN

Receiver operating characteristic (ROC) curves were used to assess the overall performance of the models. The curve depicts the sensitivity as a function of false positives (complementary to specificity). The area under the curve (AUC) is then calculated and interpreted as the probability of having the model rank a random positive example higher than a negative example [43].

Accuracy, the fraction of predictions that the model made correctly [44], is also calculated by dividing the number of correct predictions (TP and TN), among all predictions (TP, TN, FP, and FN), (Equation (6)).

(6) Accuracy=TP+TN/TP+TN+FP+FN

The free statistical software R was used for the analyses and graphics.

## 3. Results

A total of 117 patients with confirmed COVID-19 and 40 negative individuals used as controls were enrolled in this study. For the analyses, patients were categorized into four groups: men without COVID-19 (12%), women without COVID-19 (14%), men with COVID-19 (68%) and women with COVID-19 (31%). Table 1 describes the study population stratified by sex, and in general the parameters are in line with previous reports [45]. Laboratory parameters showed decreased levels of lymphocytes in COVID-19 patients, especially among men. Monocyte counts were also decreased in COVID-19 patients, but only men had a statistically significant reduction. Conversely, levels of urea were higher in COVID-19 patients, reaching statistical significance among women with COVID-19. Clinical symptoms included fever, cough, and dyspnea in COVID-19 patients regardless of sex; similarly, no statistical differences in comorbidities were found by sex.

### 3.1. Comparison between COVID-19 Patients and Non-COVID-19 (Negative Controls)

**Figure A1:**
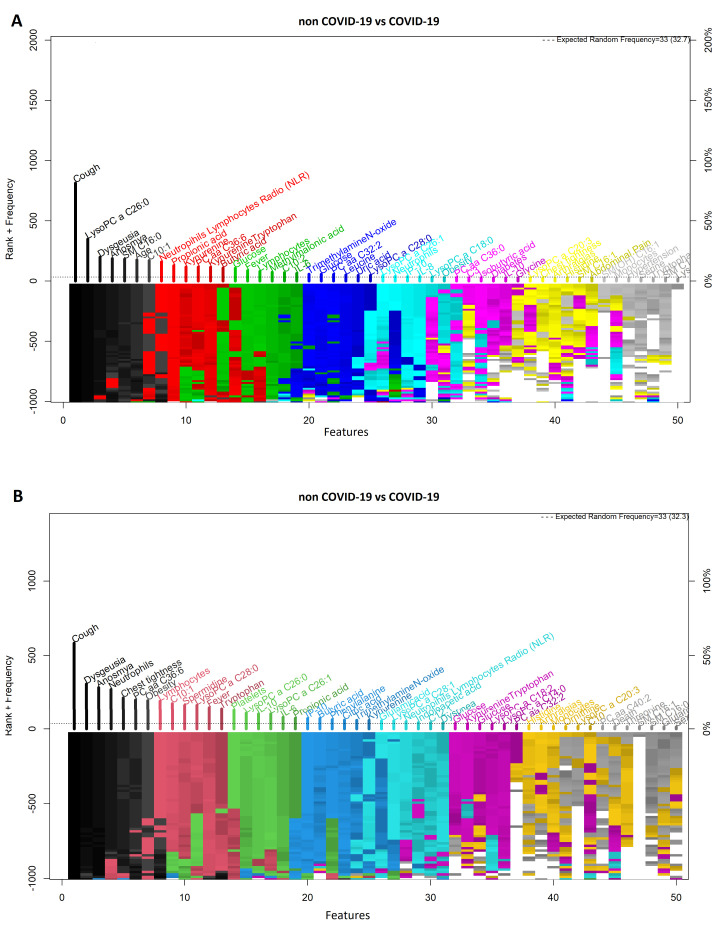
Feature–rank stability in 1000 models, for the classification of COVID-19 (**A**) Logistic regression model (**B**) Support Vector Machine model.The ”y positive” axis shows the number of times each feature was included in a given model (the frequency ranking). The ”y negative” axis shows the color coded rank of each feature as each model was generated. The ”x axis” shows the features ordered by rank. Starting color for each feature is assigned accordingly to the feature descending rank (from black down to white).

**Figure A2:**
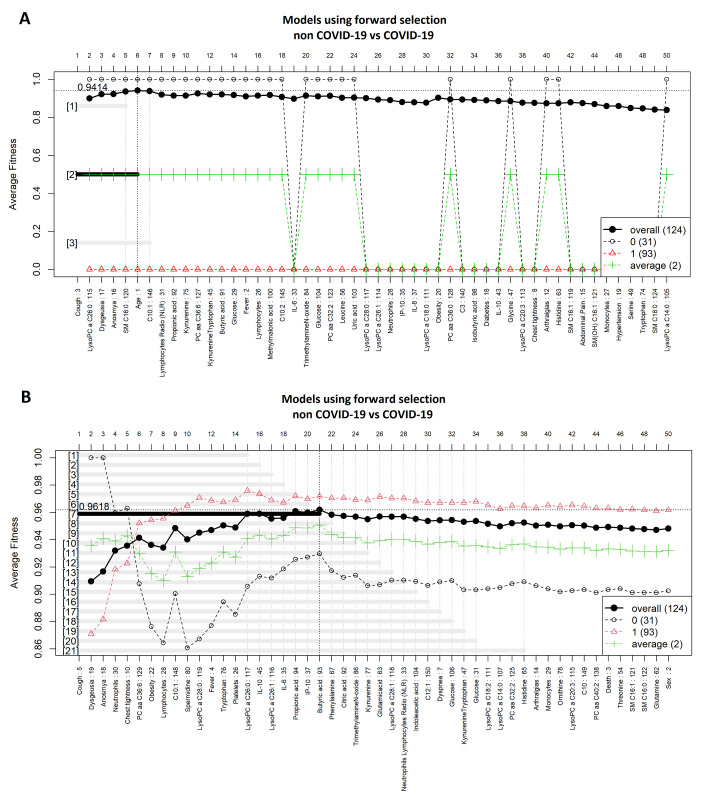
Forward selection model construction, for the prediction of COVID-19, (**A**) logistic regression and (**B**) support vector machine models. Forward selection process was performed by the most frequent feature obtained in the genetic search, then adding one feature at the time, after each feature, the accuracy of the model was obtained; if adding the feature increased the accuracy, the feature was kept. The vertical axis shows the classification accuracy. Solid line represents the overall accuracy calculated by measuring the misclassified samples divided by the total number of samples. Colored dashed lines represent the accuracy per class [36]. Note: The logistic regression function was computed using an external implementation coupled to the GALGO library; therefore, the dashed/average lines for said model could not be computed properly.

**Figure A3:**
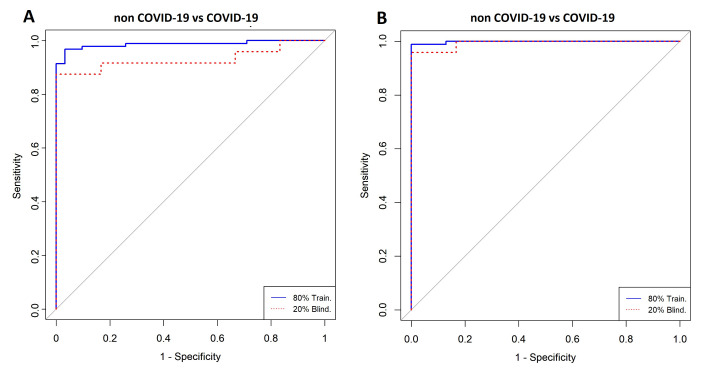
ROC curves for the prediction of COVID-19 (**A**) Logistic Regression (**B**) Support Vector Machine The model A was built with cough, lysoPC a 26:0, dysgeusia, anosmia, SM16.0, age. The model B was built with cough, dysgeusia, anosmia, neutrophils, chest pain, PC aa C36:6, obesity, lymphocytes, C10:1, spermidine, lysoPC aa C28:0, fever, tryptophan, platelets, lysoPC aa C26:0, IL-10, lysoPC aaC26:1, IL-6, propionic acid, IP-10, butyric acid.

Firstly, we analyzed the differences between COVID-19 patients and those without COVID-19, regardless of sex. Appendix A Figure A1A,B depict the ranking of variables for the LR and SVM models. The selection process was completed by entering the highest ranked variables that improved the models’ performance (Appendix A Figure A2A,B). When sex was not adjusted for, the SVM model included 21 variables. Relevant symptoms comprised cough, dysgeusia, anosmia, fever, and chest pain. Obesity was the most important comorbidity. Neutrophil, lymphocyte, and platelet counts were also relevant, as were cytokines, IL-10, IL-6, and IP-10. Metabolites included PC aa C36:6, C10:1, spermidine, lysoPC a 28:0, tryptophan, lysoPC a 26:0, lysoPC a 26:1, propionic acid, and butyric acid. For the LR model, only six variables were included: age, cough, dysgeusia, anosmia, lysoPC a 26:0, and SM C16:0. Thus, cough, anosmia, dysgeusia, and lysoPC a 26:0 were present in both models. A final model was constructed using the variables obtained from the forward selection process in both the SVM and LR algorithms. This model was then cross-validated with k=5 and blind-tested in unseen samples to assess the new samples’ performance. Appendix A Figure A3A,B depict ROC curves for both algorithms. Appendix A Table A1 shows the performance for each model with AUCs ranging from 0.93 to 0.98 for both SVM and LR.

### 3.2. Comparison of COVID-19 Status by Sex

**Figure 2:**
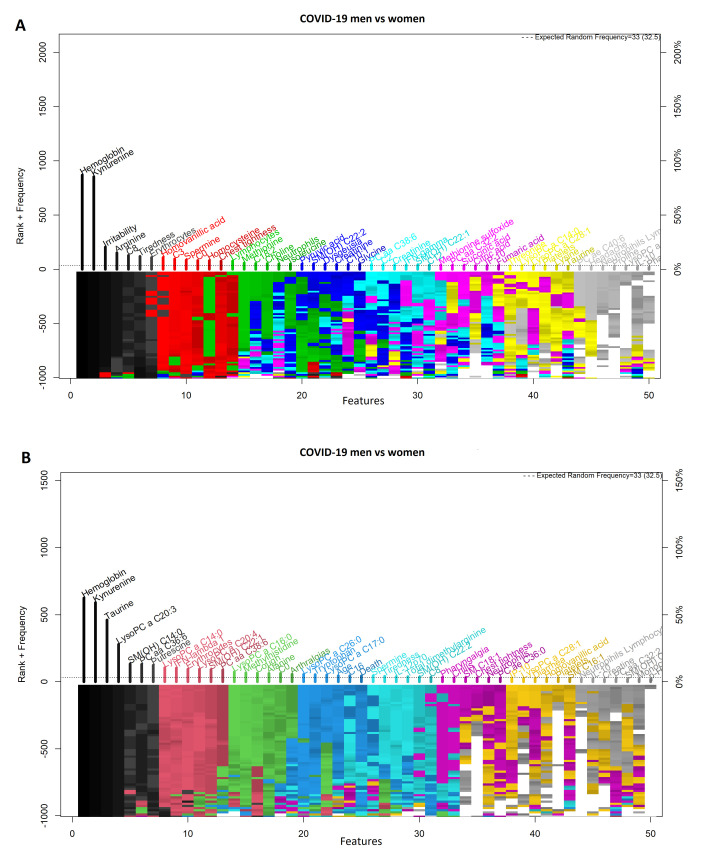
Feature–rank stability in 1000 models, for the classification of men and women with COVID-19. (**A**) Logistic regression model and (**B**) Support Vector Machine model. The ”y positive” axis shows the number of times each feature was included in a given model (the frequency ranking). The ”y negative” axis shows the color coded rank of each feature as each model was generated. The ”x” axis shows the features ordered by rank. The starting color for each feature is assigned accordingly to the feature descending rank (from black down to white).

**Figure 3:**
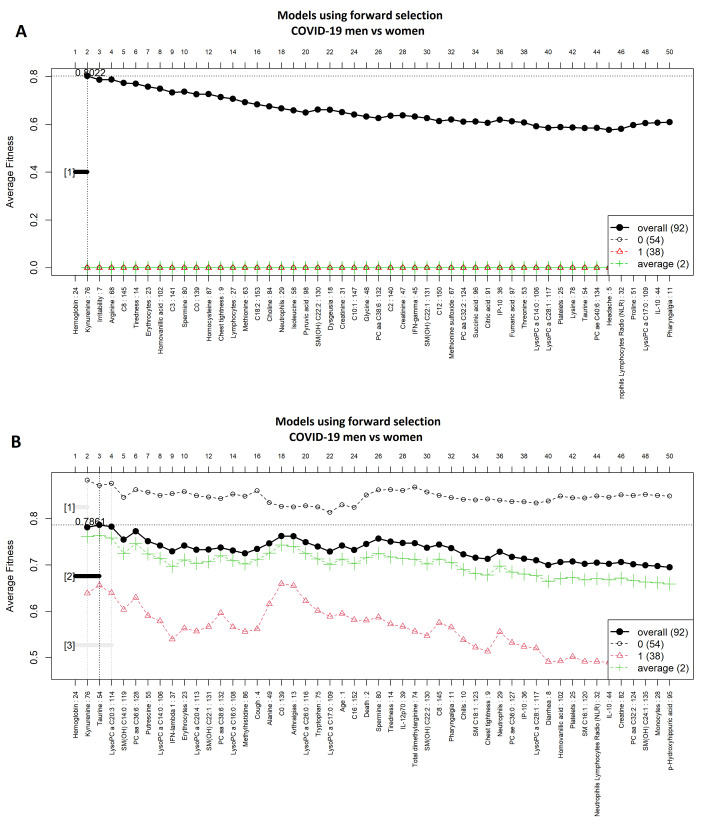
Forward selection model construction for classification of men vs. women with COVID-19. (**A**) logistic regression and (**B**) support vector machine models. The vertical axis shows the classification accuracy. The solid line represents the overall accuracy calculated by measuring the misclassified samples divided by the total number of samples. Colored dashed lines represent the accuracy per class [36]. The logistic regression function was computed using an external implementation coupled to the GALGO library; therefore, the dashed/average lines for this model could not be computed.

**Figure 4:**
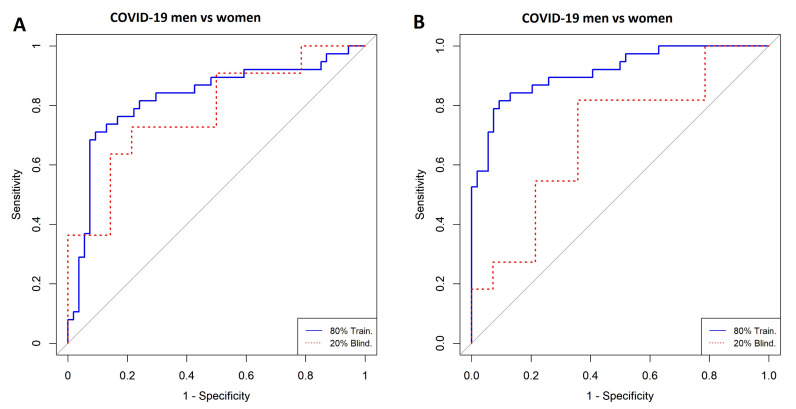
ROC curves for the classification of men vs. women with COVID-19. (**A**) Logistic Regression and (**B**) Support Vector Machine. Model A was built with the following variables: hemoglobin and kynurenine. Model B was built with hemoglobin, kynurenine, and taurine.

Figure 2 shows highly ranked variables for the LR (Figure 2A) and SVM (Figure 2B) models for men and women with a COVID-19 diagnosis. Figure 3 illustrates the forward selection process used, resulting in three variables selected in the SVM model (i.e., hemoglobin, kynurenine, and taurine), and two in the LR model (i.e., hemoglobin and kynurenine). ROCs for both models are presented in Figure 4, the ROC curves are presented for both algorithms. The model’s performance was assessed with the AUC, ranging from 0.66 to 0.94 (Table 2).

**Figure A4:**
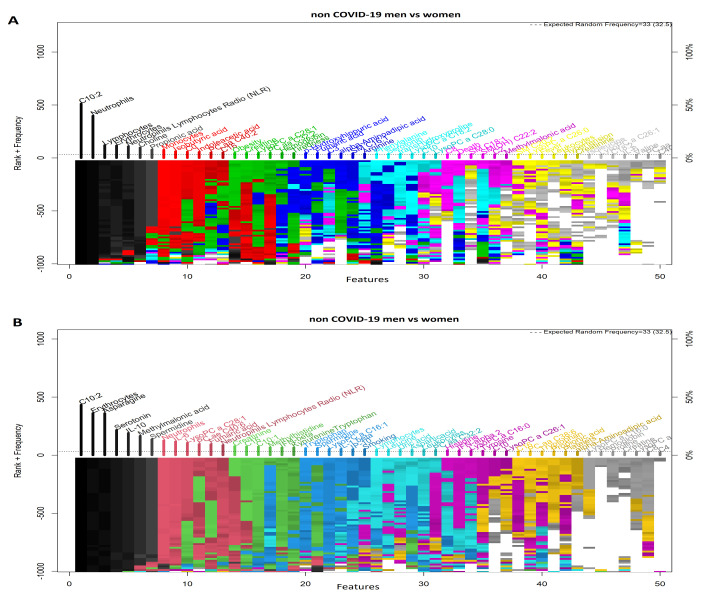
Feature–rank stability in 1000 models, for the classification of non-COVID-19 men and women (**A**) Logistic regression model (**B**) Support Vector Machine model.The ”y positive” axis shows the number of times each feature was included in a given model (the frequency ranking). The ”y negative” axis shows the color coded rank of each feature as each model was generated. The ”x” axis shows the features ordered by rank. Starting color for each feature is assigned accordingly to the feature descending rank (from black down to white).

**Figure A5:**
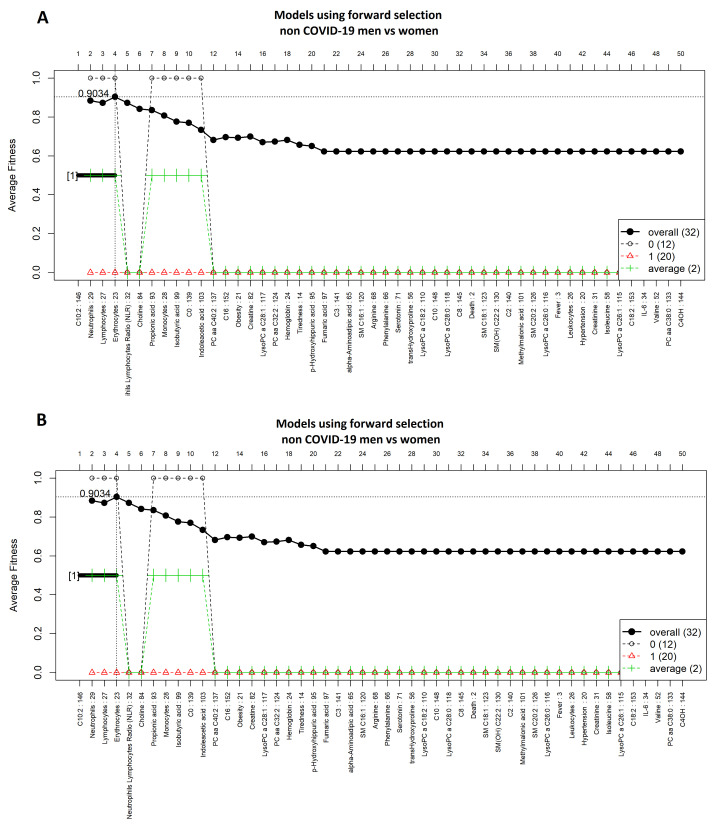
Forward selection model construction for classification of non-COVID-19 men and women (**A**) logistic regression and (**B**) support vector machine models. The vertical axis shows the classification accuracy. Solid line represents the overall accuracy calculated by measuring the misclassified samples divided by the total number of samples. Coloured dashed lines represent the accuracy per class [36]. The logistic regression function was computed using an external implementation coupled to the GALGO library; therefore, the dashed/average lines for this model could not be computed.

**Figure A6:**
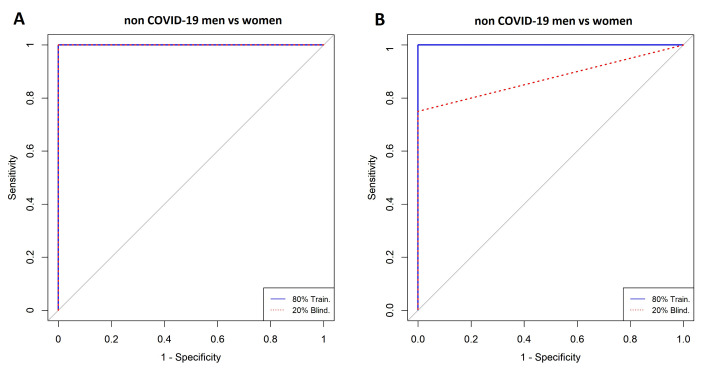
ROC curves for the classification of non-COVID-19 men and women (**A**) logistic regression and (**B**) support vector machine models.

Next, with the aim to elucidate whether kynurenine and hemoglobin were strictly in relationship with the disease, we compared men and women without COVID-19 (negative controls) by the same approach. Appendix A Figure A4 depicts highly ranked variables for the LR (Appendix A Figure A4A) and SVM (Appendix A Figure A4B) models for men and women without a COVID-19 diagnosis (i.e., control patients). Appendix A Figure A5A,B show the forward selection process used, resulting in 16 variables in the SVM model, and four in the LR model. Variables included in both models were C10:2, neutrophils, lymphocytes, and erythrocytes. ROCs are presented in Appendix A Figure A6. Appendix A Table A2 shows the AUC to assess the models’ performance that ranged from 0.66 to 1.

### 3.3. Comparison between Women with COVID-19 and Women without COVID-19

**Figure 5:**
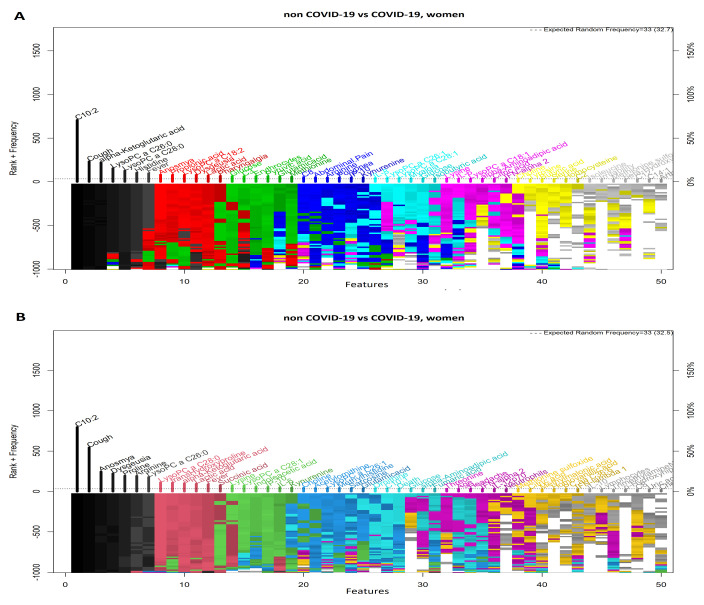
Feature–rank stability in 1000 models for the classification of women with or without COVID-19. (**A**) Logistic regression model and (**B**) Support Vector Machine model. The ”y positive” axis shows the number of times each feature was included in a given model (the frequency ranking). The ”y negative” axis shows the color coded rank of each feature as each model was generated. The ”x” axis shows the features ordered by rank. The starting color for each feature is assigned accordingly to the feature descending rank (from black down to white).

**Figure 6:**
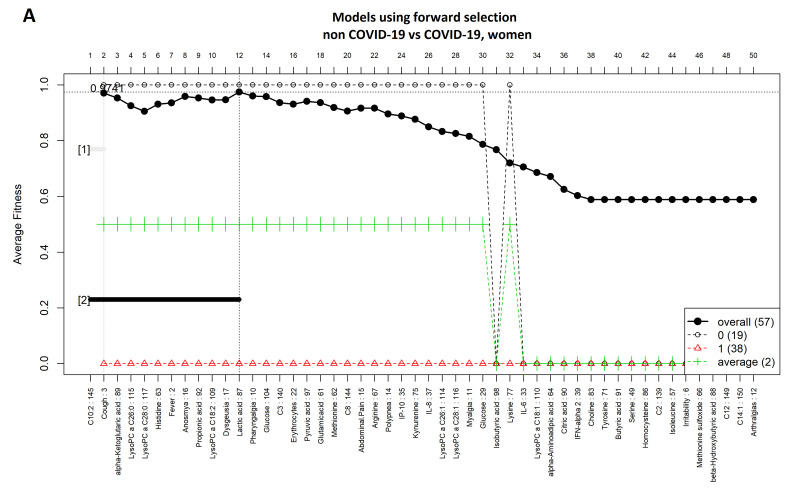

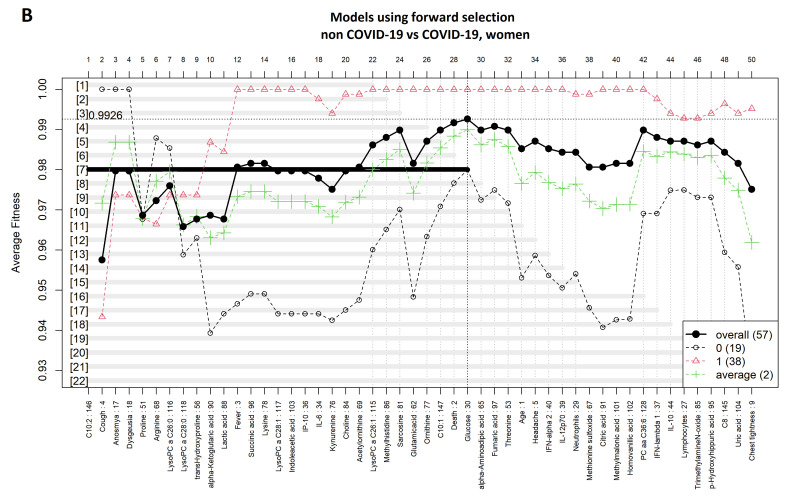
Forward selection model construction for classification of women with and without COVID-19. (**A**) logistic regression and (**B**) support vector machine models. The vertical axis shows the classification accuracy. The solid line represents the overall accuracy calculated by measuring the misclassified samples divided by the total number of samples. The colored dashed lines represent the accuracy per class [36]. The logistic regression function was computed using an external implementation coupled to the GALGO library; therefore, the dashed/average lines for this model could not be computed.

**Figure 7:**
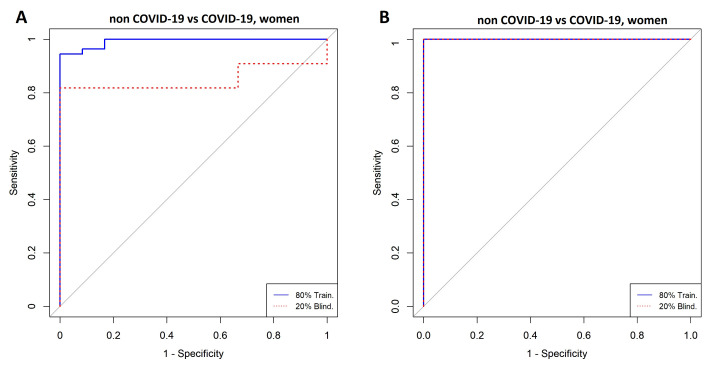
ROC curves for the classification of women with COVID-19 vs. women without COVID-19. (**A**) Logistic Regression and (**B**) Support Vector Machine. Model A was built with C10:2, cough, alpha ketoglutaric acid, lysoPC a 26:0, lysoPC a 28:0, histidine, fever, anosmia, propionic acid, lysoPC a 18:2, dysgeusia, and lactic acid. Model B was built with C10:2, cough, anosmia, dysgeusia, proline, arginine, lysoPC a C26:0, lysoPC a C28:0, transhydroxyproline, alphaketo glutaric acid, lactic acid, fever, succinic acid, lysine, lysoPC a C28:1, indoleacetic acid, IP-10, IL-6, kynurenine, choline, acethylornithine, lysoPC a C26:1, methylhistidine, sarcosine, glutamic acid, ornithine, C10:1, and glucose.

Figure 5A,B show highly ranked variables for the LR and SVM models for women with and without a COVID-19 diagnosis. The forward selection process used to identify variables is shown in Figure 6A,B, which resulted in 29 variables for the SVM model and 12 variables for the LR model. Nine variables were selected in both models (i.e., C10:2, lysoPC a C26:0, lysoPC a C28:0, alpha-ketoglutaric acid, lactic acid, cough, fever, anosmia, and dysgeusia). ROCs are shown in Figure 7A,B, and Table 3 presents the AUC to assess the models’ performance.

### 3.4. Comparison between Men with COVID-19 and Men without COVID-19

**Figure 8:**
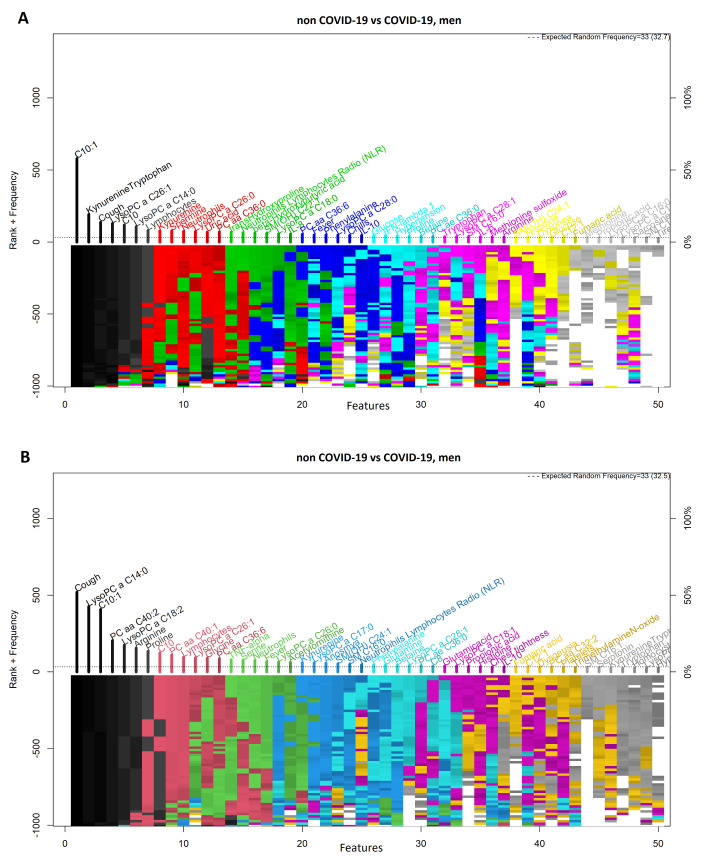
Feature–rank stability in 1000 models for the classification of men with and without COVID-19. (**A**) Logistic regression model and (**B**) Support Vector Machine model. The ”y positive” axis shows the number of times each feature was included in a given model (the frequency ranking). The ”y negative” axis shows the color coded rank of each feature as each model was generated. The ”x” axis shows the features ordered by rank. The starting color for each feature is assigned accordingly to the feature descending rank (from black down to white).

**Figure 9:**
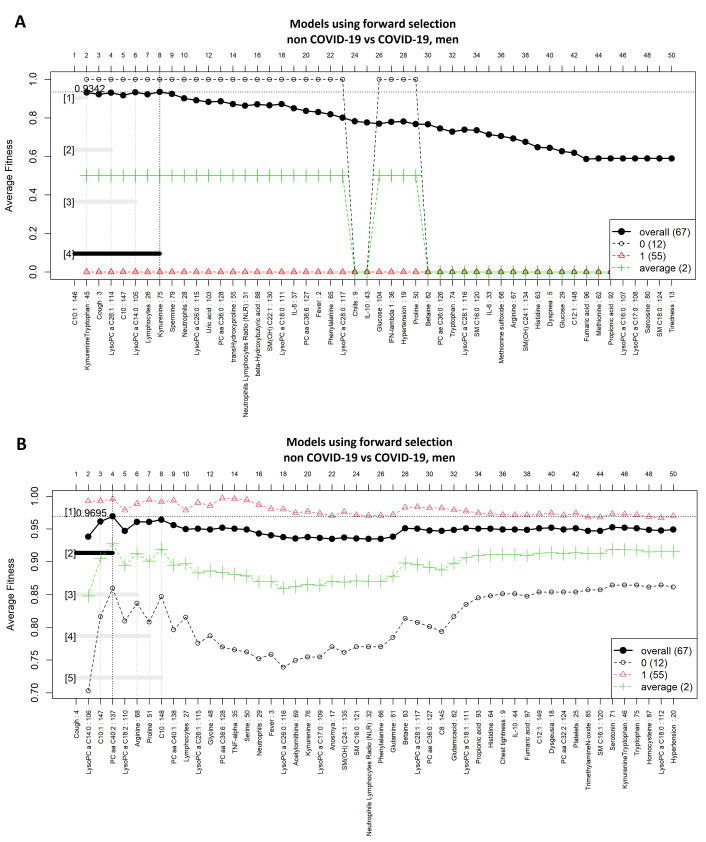
Forward selection model construction for classification of men with COVID-19 vs. men without COVID-19. (**A**) logistic regression and (**B**) support vector machine models. The vertical axis shows the classification accuracy. The solid line represents the overall accuracy calculated by measuring the misclassified samples divided by the total number of samples. The colored dashed lines represent the accuracy per class [36]. The logistic regression function was computed using an external implementation coupled to the GALGO library; therefore, the dashed/average lines for this model could not be computed.

**Figure 10:**
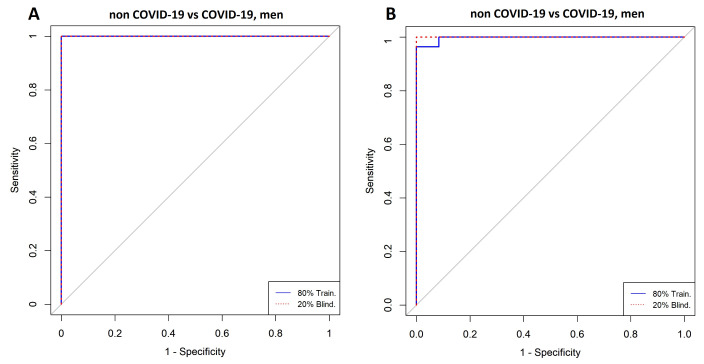
ROC curves for the classification of men with COVID-19 vs. men without COVID-19. (**A**) Logistic Regression and (**B**) Support Vector Machine. Model A was built with C10:1, kynurenine/tryptophan, cough, lysoPC a 26:1, C10, lysoPC a C14:0, lymphocytes, and kynurenine. Model B was built with cough, lysoPC a C14:0, C10:1, and PC aa C40:2.

Figure 8A,B shows highly ranked variables for the LR and SVM models, respectively, for men with and without a COVID-19 diagnosis. The forward selection for the selection process is shown in Figure 9A,B, resulting in four variables in the SVM model and eight in the LR model. Three variables were identified by both models (i.e., C10:1, cough, and LysoPC a C14:0). In Figure 10A,B the ROC curves are shown for both models. Table 4 presents the AUC to assess the performance of the models ranging from 0.96 to 1.

## 4. Discussion

Genetic algorithms and machine learning are useful tools that assist researchers and medical professionals in screening, detecting, and predicting several diseases, including COVID-19. Here, they were used to identify potential sex differences associated with COVID-19 using numerous symptoms, metabolites, and cytokines measured in 157 individuals. The genetic algorithm was used to build a multivariate model able to predict and classify COVID-19 patients along with two machine learning algorithms (SVM and LR), extensively tested for classification tasks [46,47,48,49,50]. One of the key advantages of the proposed methodology, is that a genetic algorithm searches for the combined classification power rather than for individual performance of each feature (Table 1).

When comparing COVID-19 patients with control individuals without sex stratification, cough, anosmia, dysgeusia, and lysoPC a 26:0 were identified by SVM and LR, with a similar performance and sensitivity, even though the former required 21 variables instead only 6 as the latter. Zoabi and colleagues also developed a model for predicting COVID-19 using machine learning that included fever and cough as the most important symptoms [51]. Similarly, Tandan and colleagues found that fever, cough, pneumonia, and sore throat were the most frequent features using a rule-based machine learning technique called association rule mining [52]. Other authors have reported lipid dysregulations in COVID-19 patients, as found in this study, such as that of glycerophospholipid metabolism [53,54,55].

Studies of COVID-19 patients have shown that men have a higher risk of developing severe illness compared to women, as well as fatalities [56]. The underlying mechanisms for such differences, reflected also in clinical symptoms, metabolic alterations, and immune response, still remain insufficiently understood. Therefore, more research should focus on the role of sex as a relevant factor in COVID-19. The method used was GALGO [57], an R package for multivariate selection technique based on genetic algorithms that produce thousands of models’ combinations keeping only the best variables in the final model. Here, hemoglobin plus kynurenine and hemoglobin in combination drove the differences between men and women with COVID-19. The inclusion of hemoglobin should not be surprising, even when levels fell within normal range in this study, as others have reported a significant drop in hemoglobin values associated with disease severity [57]. Other authors have also reported remarkably low levels of hemoglobin and albumin in COVID-19 patients, probably due to the rapid turnover of red blood cells that led to hemoglobin degradation [58]. So, apart from the classic pulmonary immune-inflammation explanation, the occurrence of an oxygen-deprived blood disease (i.e., hemoglobinopathies with iron metabolism dysregulation) appears to be playing a major role in the pathophysiology of this infection [59]. From the metabolic point of view, the simultaneous inclusion of hemoglobin and kynurenine can be expected. Kynurenine is a metabolic result of tryptophan degradation. In COVID-19 patients, there is a decrease in tryptophan levels and an increase in kynurenine compared with non-infected individuals, and there is a positive correlation between tryptophan levels and hemoglobin in men and women with COVID-19. The mechanism behind this in COVID-19 individuals is an increase in indoleamine 2,3-dioxygenase activity. Cytokine-induced (i.e., interferon-γ and tumor necrosis factor-α) tryptophan degradation via this enzyme suppresses erythropoiesis. Tryptophan is a nutritional pyrrole source essential for hemoglobin synthesis, and therefore the enhanced degradation of tryptophan is involved in a hemoglobin drop of blood levels and in the further development of anemia [60,61]. On the other hand, the kynurenine pathway plays a crucial role in the regulation of the immune response, notably as a counter-regulatory mechanism in the context of inflammation. It has been seen that the immune response against SARS-CoV-2 is different between men and women [62]. A previous article has linked metabolic markers with these immune differences in both sexes (i.e., kynurenic acid, another metabolite involved in the kynurenine pathway) [12]; the authors found that kynurenic acid correlated well with several immune markers only in male patients. In conclusion, the proposed model to address differences between men and women with COVID-19 also detected one metabolite belonging to the kynurenine pathway. Metabolites in the kynurenine pathway are critical immunomodulators, contributing to immunosuppressive activity of dendritic cells and to CD8+ T cell suppression. Therefore, activating this pathway may allow SARS-CoV-2 to evade immunity [63]. The differentiated expression of kynurenine in men and women could be interpreted as a different activation of IDO, which in turn is a consequence of IFNs and cytokines production. A recent study reported that after TLR7 stimulation, IFN levels were lower in men compared with women. Toll-like receptor 7–mediated IFN expression may be decreased in men due to the known negative effects of testosterone on IFN expression [64]. Sex-disaggregated data are not widely available, and consistent reporting of data based on sex is limited at the moment. We provide here predictive models for men and women, since we demonstrated that there are sex-specific differences in COVID-19 patients. The model that included C10:2, lysoPC a 26:0, lysoPC a 28:0, alpha ketoglutaric acid, lactic acid, cough, fever, anosmia, and dysgeusia differentiated well between women with COVID-19 and women without COVID-19, with some of these being reported in earlier studies; for instance, anosmia and dysgeusia have been found more frequently in women than in men [33]. Between men without COVID-19 and men with COVID-19, cough and metabolites LysoPC a 14:0 and C10:1 were the predictors included in the models. In an earlier study, lysophosphatidylcholine (14:0) was reported to be negatively associated with COVID-19 [65,66]. Decreased plasma lysophosphatidylcholines (LPCs) levels have been associated with unfavorable disease outcomes, such as sepsis mortality and hospital mortality in patients with pneumonia [67]. LPCs have been recognized as important homeostatic mediators involved in inflammation and the activation of immune cells. Furthermore, they have been found to act as strong chemo-attractants for monocytes, T cells, as well as natural killer (NK) cells, drawing them to inflammation sites [67]. Lysophosphatidylcholine (14:0) was found negatively associated with COVID-19 in a study performed by Cai et al. [12]. None of the models proposed by us to differentiate between men with COVID-19 and men without COVID-19 found cytokines/chemokines as important features, while in women, SVM identified IP-10 and IL-6 as important features to discriminate between those with COVID-19 and those without. This may be further evidence about the sex-related differential immune activation.

Studies agree that male sex is a strong risk factor for increased mortality, together with other factors such as circulating hormones, ACE receptors, immune incompetence, older age, and comorbidities such as diabetes and cardiovascular disease [68,69]. Identifying the mechanisms of sexual dimorphism in SARS-CoV-2 could thus provide important information about the physiopathology of COVID-19.

It was observed that the models’ performance dropped when tested in blind tests with “new patients”. Moreover, when blind-tested, SVM performance was weaker than LR, indicating a tendency to overfit the data. Blind testing showed that the best metrics were obtained when LR was used, as this method builds models with fewer variables and higher statistical significance. Conversely, SVM showed higher performance in terms of model accuracy. Yet, both models maintained statistical power to differentiate and classify COVID-19 patients. Considering accuracy as an important criterion for comparing the performance of models in this domain, it can be argued that SVM has better efficiency than LR. However, regardless of the classification model used, statistically significant metrics and appropriate performances were achieved.

## 5. Conclusions

There are clinical and metabolic differences between men and women with COVID-19. The machine learning classification methods used show statistically significant variables that predicted specific sex characteristics. This work reinforces the need to take into account the sex differences to accurately diagnose COVID-19 infection. A combination of kynurenine and hemoglobin could, for instance, help discriminate between infected men and women, revealing underlying metabolic and hematologic differences associated with biological sex. We found that different metabolites are needed to discern COVID-19 in women and in men. In women, a panel consisting of C10:2, lysoPC a 26:0, lysoPC a 28:0, alpha ketoglutaric acid, lactic acid, cough, fever, anosmia, and dysgeusia differentiated well between women with and women without COVID-19. In men, cough, LysoPC a 14:0, and C10:1 were the most useful variables to differentiate COVID-19. However, we have to acknowledge some limitations. One limitation is the cross-sectional exploratory nature of the study design. This design prevented a longitudinal metabolite assessment as well as monitoring of clinical and hematological perturbations. Furthermore, we did not measure sex hormones, which may have a possible role in the differences found between COVID-19 women and men. Furthermore, due to the lack of collected data, the impact of gender was not analyzed by us.

## Figures and Tables

**Table 1:** Sociodemographic, epidemiological, and clinical characteristics, including laboratory analyses, of the study participants by sex.

	Non-COVID-19			COVID-19		
**Variable**	**Men**	**Women**	**p Value**	**Men**	**Women**	**p Value**
Age	40 (37–53)	40 (37–46)	0.59	53 (42–63)	58 (52–62)	0.11
**Lab Data**						
Erythrocytes (million/mL)	5.5 ± 0.4	4.9 ± 0.3	<0.0001	5.4 (5.1–5.7)	4.9 ± 0.6	<0.0001
Hemoglobin (g/dL)	16.3 (15.5–17)	14.7 ± 0.9	<0.0001	16.1 (15.3–16.7)	13.9 ± 1.9	<0.0001
Platelets (thousands/mL)	260.7 ± 58.5	297.2 ± 65.9	0.03	262.6 ± 94.9	227.4 ± 74.7	0.07
Leukocytes (×10${}^{3}$)	7.3 ± 2.1	7.3 ± 2.3	0.84	8.4 (5.7–10)	7.4 (5.3–10.6)	0.67
Lymphocytes (%)	33 ± 9.9	28.1 ± 8	0.05	9.7 (6–16.3)	15.1 (10.3–26.4)	0.0006
Monocytes (%)	7.5 ± 2.3	6.2 ± 2.5	0.2	4.8 (2.9–7.2)	4.5 (3–7.6)	0.92
Neutrophils (%)	55.4 (50.5–61)	63.2 ± 9	0.04	84.8 (72.9–89.5)	78.5 (66.7–84.8)	0.02
Glucose	93.2 ± 13.6	93.5 (84.3–106.8)	0.79	119 (96.5–140.5)	121 (97–264)	0.27
Creatinine	1 ± 0.16	0.8 (0.7–0.9)	0.0009	0.95 (0.8–1.2)	0.8 (0.7–1)	0.003
Urea	32.8 (27.9–39.9)	29.1 ± 9.7	0.12	33.6 (25.4–42.5)	49.6 (32–76.7)	0.008
**Variable**	**Men**	**Women**	**p Value**	**Men**	**Women**	**p Value**
**Symptomatology, n (%)**						
Fever	0 (0)	0 (0)	>0.9	42 (61.8)	27(55.1)	0.57
Cough	0 (0)	0 (0)	>0.9	57 (83.8)	38 (77.6)	0.47
Headache	12 (66.7)	17 (77)	0.49	41 (60.3	33 (67.4)	0.56
Dyspnoea	2 (11.1)	3 (13.6)	>0.9	47 (69.1)	29 (59.2)	0.33
Irritability	1 (5.6)	2 (9)	>0.9	4 (5.9)	3 (6.1)	>0.9
Diarrhea	1 (6.6)	1 (4.6)	>0.9	10 (14.7)	8 (16.3)	0.8
Chest tightness	0 (0)	2 (9.1)	0.49	18 (26.5)	15 (30.6)	0.68
Chills	2 (11.1)	3 (12.6)	>0.9	28 (41.2)	18 (36.7)	0.7
Pharyngalgia	7 (38.9)	10 (45.95	0.75	30 (44.1)	16 (32.7)	0.25
Myalgia	7 (38.9)	8 (36.4)	>0.9	33 (48.5)	33 (67.4)	0.06
Arthralgias	4 (22.2)	7 (31.8)	0.72	32 (47.1)	32 (65.3)	0.06
Rhinorrhea	1 (5.6)	5 (22.7)	0.19	12 (17.9)	9 (18.4)	>0.9
Polypnea	0 (0)	1 (4.6)	>0.9	6 (8.8)	7 (14.3)	0.38
Vomiting	0 (0)	0 (0)	>0.9	4 (5.9)	5 (10.2)	0.49
Abdominal pain	2 (11.1)	2 (9.1)	>0.9	8 (10.5)	5 (10.2)	>0.9
Conjunctivitis	0 (0)	1 (4.6)	>0.9	2 (2.9)	1 (2)	>0.9
Cyanosis	0 (0)	1 (4.6)	>0.9	0 (0)	1 (2)	0.42
Anosmya	0 (0)	0 (0)	>0.9	10 (14.7)	13 (26.5)	0.16
Dysgeusia	0 (0)	0 (0)	>0.9	10 (14.7)	14 (28.6)	0.1
**Comorbidities (self-reported), n (%)**						
Diabetes	1 (5.6)	2 (9.1)	>0.9	15 (22.1)	18 (36.7)	0.09
Hypertension	5 (27.8)	4 (18.2)	0.71	26 (38.8)	18 (36.7)	0.85
COPD	0 (0)	0 (0)	>0.9	1 (1.5)	0 (0)	>0.9
Asthma	2 (11.1)	0 (0)	0.19	0 (0)	2 (4.1)	0.17
Immunosuppression	0 (0)	2 (9.1)	0.49	1 (1.5)	0 (0)	>0.9
HIV/AIDS	1 (5.6)	0 (0)	0.45	0 (0)	0 (0)	>0.9
Cardiovascular disease	0 (0)	0 (0)	>0.9	2 (2.9)	1 (2)	>0.9
Obesity (>30 kg/m^2^)	1 (5.6)	2 (9.1)	>0.9	17 (25)	14 (28.6)	0.68
Chronic renal insufficiency	1 (5.6)	0 (0)	0.45	3 (4.4)	1 (2)	0.64
Smoking	2 (11.1)	2 (9.1)	>0.9	6 (8.8)	1 (2)	0.24
**Treatment (self-reported), n (%)**						
Antipyretics	3 (16.7)	1 (4.6)	0.31	20 (30.3)	14 (28.6)	>0.9

**Table 2:** Evaluation of performance for LR and SVM models for male and female COVID-19 patients.

Men with COVID-19 vs. Women with COVID-19		Model	
		SVM	LR
Included Variables		3	2
Cross-validation (k = 5)	AUC	0.91	0.82
	CI 95%	0.86–0.98	0.71–0.92
	Specificity	0.94	0.90
	Sensitivity	0.80	0.73
	Accuracy	0.88	0.83
Training (80%)	AUC	0.92	0.82
	CI 95%	0.85–0.98	0.73–0.92
	Specificity	0.92	0.90
	Sensitivity	0.84	0.71
	Accuracy	0.89	0.82
Blind (20%)	AUC	0.66	0.77
	CI 95%	0.47–0.91	0.58–0.97
	Specificity	0.71	0.78
	Sensitivity	0.72	0.72
	Accuracy	0.72	0.76

**Table 3:** Evaluation criteria for the logistic regression model with all factors and support vector machine for women with COVID-19 vs. women without COVID-19.

Women with COVID-19 vs. Women without COVID-19		Model	
		SVM	LR
Included Variables		29	12
Cross-validation (k = 5)	AUC	1	0.99
	CI 95%	1–1	0.96–1
	Specificity	1	1
	Sensitivity	1	1
	Accuracy	1	1
Training (80%)	AUC	1	1
	CI 95%	1–1	0.97–1
	Specificity	1	1
	Sensitivity	1	1
	Accuracy	1	1
Blind (20%)	AUC	1	0.85
	CI 95%	1–1	0.64–1
	Specificity	1	1
	Sensitivity	1	1
	Accuracy	1	1

**Table 4:** Evaluation criteria for logistic regression model with all factors and support vector machine for men with COVID-19 vs. men without COVID-19.

Men with COVID-19 vs. Men without COVID-19		Model	
		SVM	LR
Included Variables		4	8
Cross-validation (k = 5)	AUC	0.99	1
	CI 95%	0.99–1	1–1
	Specificity	1	1
	Sensitivity	0.97	1
	Accuracy	0.97	1
Training (80%)	AUC	0.99	1
	CI 95%	0.98–1	1–1
	Specificity	1	1
	Sensitivity	0.96	1
	Accuracy	0.97	1
Blind (20%)	AUC	1	1
	CI 95%	1–1	1–1
	Specificity	1	1
	Sensitivity	1	1
	Accuracy	1	1

## Data Availability

The data presented in this study are openly available in Mendeley at doi: 10.17632/x9tw3knwsd.1.

## Appendix A

### Appendix A.1

**Table A1:** Evaluation criteria for logistic regression model with all factors and support vector machine for COVID-19 and non COVID-19 without considering sex.

COVID-19 vs. Non COVID-19		Model	
		SVM	LR
Included Variables		21	6
Cross-Validation (k = 5)	AUC	0.99	0.98
	CI 95%	0.99–1	0.96–1
	Specificity	1	0.97
	Sensitivity	0.99	0.95
	Accuracy	0.99	0.96
Training (80%)	AUC	0.99	0.98
	CI 95%	0.99–1	0.96-1
	Specificity	1	0.96
	Sensitivity	0.98	0.96
	Accuracy	0.99	0.96
Blind (20%)	AUC	0.99	0.93
	CI 95%	0.97–1	0.93-1
	Specificity	1	1
	Sensitivity	0.95	0.87
	Accuracy	0.96	0.9

**Table A2:** Evaluation criteria for logistic regression model with all factors and support vector machine for non-COVID-19 men and women patients.

Non COVID-19 Men vs. COVID-19 Women		Model	
		SVM	LR
Included Variables		16	4
Cross Validation (k = 5)	AUC	1	1
	CI 95%	1–1	1–1
	Specificity	1	1
	Sensitivity	1	1
	Accuracy	1	1
Training (80%)	AUC	1	1
	CI 95%	1–1	1–1
	Specificity	1	1
	Sensitivity	1	1
	Accuracy	1	1
Blind (20%)	AUC	0.83	1
	CI 95%	0.76–0.90	1–1
	Specificity	0.66	1
	Sensitivity	1	1
	Accuracy	0.8	1

## References

[1] Ghosh S., Klein R.S. (2017). Sex Drives Dimorphic Immune Responses to Viral Infections. J. Immunol..

[2] Klein S.L., Flanagan K.L. (2016). Sex differences in immune responses. Nat. Rev. Immunol..

[3] Karlberg J., Chong D.S., Lai W.Y. (2004). Do men have a higher case fatality rate of severe acute respiratory syndrome than women do?. Am. J. Epidemiol..

[4] Matsuyama R., Nishiura H., Kutsuna S., Hayakawa K., Ohmagari N. (2016). Clinical determinants of the severity of Middle East respiratory syndrome (MERS): A systematic review and meta-analysis. BMC Public Health.

[5] Eshima N., Tokumaru O., Hara S., Bacal K., Korematsu S., Tabata M., Karukaya S., Yasui Y., Okabe N., Matsuishi T. (2011). Sex- and age-related differences in morbidity rates of 2009 pandemic influenza A H1N1 virus of swine origin in Japan. PLoS ONE.

[6] Peckham H., de Gruijter N.M., Raine C., Radziszewska A., Ciurtin C., Wedderburn L.R., Rosser E.C., Webb K., Deakin C.T. (2020). Male sex identified by global COVID-19 meta-analysis as a risk factor for death and ITU admission. Nat. Commun..

[7] Ten-Caten F., Gonzalez-Dias P., Castro I., Ogava R.L.T., Giddaluru J., Silva J.C.S., Martins F., Goncalves A.N.A., Costa-Martins A.G., Araujo J.D. (2021). In-depth analysis of laboratory parameters reveals the interplay between sex, age, and systemic inflammation in individuals with COVID-19. Int. J. Infect. Dis..

[8] Ding T., Zhang J., Wang T., Cui P., Chen Z., Jiang J., Zhou S., Dai J., Wang B., Yuan S. (2021). Potential Influence of Menstrual Status and Sex Hormones on Female Severe Acute Respiratory Syndrome Coronavirus 2 Infection: A Cross-sectional Multicenter Study in Wuhan, China. Clin. Infect. Dis..

[9] Rastrelli G., Di Stasi V., Inglese F., Beccaria M., Garuti M., Di Costanzo D., Spreafico F., Greco G.F., Cervi G., Pecoriello A. (2021). Low testosterone levels predict clinical adverse outcomes in SARS-CoV-2 pneumonia patients. Andrology.

[10] Chang E., Varghese M., Singer K. (2018). Gender and Sex Differences in Adipose Tissue. Curr. Diab. Rep..

[11] Karastergiou K., Fried S.K. (2017). Cellular Mechanisms Driving Sex Differences in Adipose Tissue Biology and Body Shape in Humans and Mouse Models. Adv. Exp. Med. Biol..

[12] Cai Y., Kim Daniel J., Takahashi T., Broadhurst David I., Yan H., Ma S., Rattray Nicholas J.W., Casanovas-Massana A., Israelow B., Klein J. (2021). Kynurenic acid may underlie sex-specific immune responses to COVID-19. Sci. Signal..

[13] Dix A., Vlaic S., Guthke R., Linde J. (2016). Use of systems biology to decipher host–pathogen interaction networks and predict biomarkers. Clin. Microbiol. Infect..

[14] Hou Q., Bing Z.T., Hu C., Li M.Y., Yang K.H., Mo Z., Xie X.W., Liao J.L., Lu Y., Horie S. (2018). RankProd Combined with Genetic Algorithm Optimized Artificial Neural Network Establishes a Diagnostic and Prognostic Prediction Model that Revealed C1QTNF3 as a Biomarker for Prostate Cancer. EBioMedicine.

[15] Xie Y., Meng W.Y., Li R.Z., Wang Y.W., Qian X., Chan C., Yu Z.F., Fan X.X., Pan H.D., Xie C. (2021). Early lung cancer diagnostic biomarker discovery by machine learning methods. Transl. Oncol..

[16] Cristianini N., Ricci E. (2008). Support Vector Machines. Encyclopedia of Algorithms.

[17] Velazquez-Pupo R., Sierra-Romero A., Torres-Roman D., Shkvarko Y.V., Santiago-Paz J., Gomez-Gutierrez D., Robles-Valdez D., Hermosillo-Reynoso F., Romero-Delgado M. (2018). Vehicle Detection with Occlusion Handling, Tracking, and OC-SVM Classification: A High Performance Vision-Based System. Sensors.

[18] Gao L., Ren Z., Tang W., Wang H., Chen P. (2010). Intelligent gearbox diagnosis methods based on SVM, wavelet lifting and RBR. Sensors.

[19] Ruiz-Gonzalez R., Gomez-Gil J., Gomez-Gil F.J., Martinez-Martinez V. (2014). An SVM-based classifier for estimating the state of various rotating components in agro-industrial machinery with a vibration signal acquired from a single point on the machine chassis. Sensors.

[20] Men H., Fu S., Yang J., Cheng M., Shi Y., Liu J. (2018). Comparison of SVM, RF and ELM on an Electronic Nose for the Intelligent Evaluation of Paraffin Samples. Sensors.

[21] Dreiseitl S., Ohno–Machado L. (2002). Logistic regression and artificial neural network classification models: A methodology review. J. Biomed. Inform..

[22] Lessmann S., Stahlbock R., Crone S.F. Genetic Algorithms for Support Vector Machine Model Selection. Proceedings of the 2006 International Joint Conference on Neural Networks.

[23] Dalal V., Carmicheal J., Dhaliwal A., Jain M., Kaur S., Batra S.K. (2020). Radiomics in stratification of pancreatic cystic lesions: Machine learning in action. Cancer Lett..

[24] Xu W., Xu M., Wang L., Zhou W., Xiang R., Shi Y., Zhang Y., Piao Y. (2019). Integrative analysis of DNA methylation and gene expression identified cervical cancer-specific diagnostic biomarkers. Signal Transduct. Target Ther..

[25] Chang C.H., Lin C.H., Lane H.Y. (2021). Machine Learning and Novel Biomarkers for the Diagnosis of Alzheimer’s Disease. Int. J. Mol. Sci..

[26] Manoochehri Z., Salari N., Rezaei M., Khazaie H., Manoochehri S., Pavah B.K. (2018). Comparison of support vector machine based on genetic algorithm with logistic regression to diagnose obstructive sleep apnea. J. Res. Med. Sci..

[27] Guhathakurata S., Kundu S., Chakraborty A., Banerjee J.S. (2021). A novel approach to predict COVID-19 using support vector machine. Data Science for COVID-19.

[28] Jiang X., Coffee M., Bari A., Wang J., Jiang X., Huang J., Shi J., Dai J., Cai J., Zhang T. (2020). Towards an Artificial Intelligence Framework for Data-Driven Prediction of Coronavirus Clinical Severity. Comput. Mater. Contin..

[29] Xu W., Sun N.N., Gao H.N., Chen Z.Y., Yang Y., Ju B., Tang L.L. (2021). Risk factors analysis of COVID-19 patients with ARDS and prediction based on machine learning. Sci. Rep..

[30] Lu J.Q., Musheyev B., Peng Q., Duong T.Q. (2021). Neural network analysis of clinical variables predicts escalated care in COVID-19 patients: A retrospective study. PeerJ..

[31] Li X., Ge P., Zhu J., Li H., Graham J., Singer A., Richman P.S., Duong T.Q. (2020). Deep learning prediction of likelihood of ICU admission and mortality in COVID-19 patients using clinical variables. PeerJ.

[32] Hou W., Zhao Z., Chen A., Li H., Duong T.Q. (2021). Machining learning predicts the need for escalated care and mortality in COVID-19 patients from clinical variables. Int. J. Med Sci..

[33] Ancochea J., Izquierdo J.L., Soriano J.B. (2021). Evidence of Gender Differences in the Diagnosis and Management of Coronavirus Disease 2019 Patients: An Analysis of Electronic Health Records Using Natural Language Processing and Machine Learning. J. Women’s Health.

[34] Zheng J., Zhang L., Johnson M., Mandal R., Wishart D.S. (2020). Comprehensive Targeted Metabolomic Assay for Urine Analysis. Anal. Chem..

[35] Curtis A.E., Smith T.A., Ziganshin B.A., Elefteriades J.A. (2016). The mystery of the Z-score. Aorta.

[36] Trevino V., Falciani F. (2006). GALGO: An R package for multivariate variable selection using genetic algorithms. Bioinformatics.

[37] Couronné R., Probst P., Boulesteix A.L. (2018). Random forest versus logistic regression: A large-scale benchmark experiment. BMC Bioinform..

[38] Chakravarthi B.R., Priyadharshini R., Muralidaran V., Jose N., Suryawanshi S., Sherly E., McCrae J.P. (2021). DravidianCodeMix: Sentiment Analysis and Offensive Language Identification Dataset for Dravidian Languages in Code-Mixed Text. arXiv.

[39] Kleinbaum D.G., Klein M. (2010). Introduction to logistic regression. Logistic Regression.

[40] Zou X., Hu Y., Tian Z., Shen K. (2019). Logistic regression model optimization and case analysis. Proceedings of the 2019 IEEE 7th International Conference on Computer Science and Network Technology (ICCSNT), Dalian, China, 19–20 October 2019.

[41] Suthaharan S. (2016). Machine learning models and algorithms for big data classification. Integr. Ser. Inf. Syst.

[42] Miller A. (2002). Subset Selection in Regression.

[43] Rakotomamonjy A. Optimizing Area Under Roc Curve with SVMs. Proceedings of the Conference: ROC Analysis in Artificial Intelligence, 1st International Workshop, ROCAI-2004.

[44] Yin M., Wortman Vaughan J., Wallach H. Understanding the effect of accuracy on trust in machine learning models. Proceedings of the 2019 CHI Conference on Human Factors in computing Systems.

[45] Wang M., Jiang N., Li C., Wang J., Yang H., Liu L., Tan X., Chen Z., Gong Y., Yin X. (2021). Sex-Disaggregated Data on Clinical Characteristics and Outcomes of Hospitalized Patients with COVID-19: A Retrospective Study. Front. Cell. Infect. Microbiol..

[46] Feng J.z., Wang Y., Peng J., Sun M.w., Zeng J., Jiang H. (2019). Comparison between logistic regression and machine learning algorithms on survival prediction of traumatic brain injuries. J. Crit. Care.

[47] Li Y., Chen Z. (2018). Performance evaluation of machine learning methods for breast cancer prediction. Appl. Comput. Math..

[48] Kang J., Schwartz R., Flickinger J., Beriwal S. (2015). Machine learning approaches for predicting radiation therapy outcomes: A clinician’s perspective. Int. J. Radiat. Oncol. Biol. Phys..

[49] Ayon S.I., Islam M.M., Hossain M.R. (2020). Coronary artery heart disease prediction: A comparative study of computational intelligence techniques. IETE J. Res..

[50] Liang M., Cai Z., Zhang H., Huang C., Meng Y., Zhao L., Li D., Ma X., Zhao X. (2019). Machine learning-based analysis of rectal cancer MRI radiomics for prediction of metachronous liver metastasis. Acad. Radiol..

[51] Zoabi Y., Deri-Rozov S., Shomron N. (2021). Machine learning-based prediction of COVID-19 diagnosis based on symptoms. Npj Digit. Med..

[52] Tandan M., Acharya Y., Pokharel S., Timilsina M. (2021). Discovering symptom patterns of COVID-19 patients using association rule mining. Comput. Biol. Med..

[53] Fraser D.D., Slessarev M., Martin C.M., Daley M., Patel M.A., Miller M.R., Patterson E.K., O’Gorman D.B., Gill S.E., Wishart D.S. (2020). Metabolomics profiling of critically ill coronavirus disease 2019 patients: Identification of diagnostic and prognostic biomarkers. Crit. Care Explor..

[54] Delafiori J., Navarro L.C., Siciliano R.F., De Melo G.C., Busanello E.N.B., Nicolau J.C., Sales G.M., De Oliveira A.N., Val F.F.A., De Oliveira D.N. (2021). COVID-19 automated diagnosis and risk assessment through metabolomics and machine learning. Anal. Chem..

[55] Sindelar M., Stancliffe E., Schwaiger-Haber M., Anbukumar D.S., Albrecht R.A., Adkins-Travis K., Garcia-Sastre A., Shriver L.P., Patti G.J. (2021). Longitudinal metabolomics of human plasma reveals robust prognostic markers of COVID-19 disease severity. medRxiv.

[56] Dana P.M., Sadoughi F., Hallajzadeh J., Asemi Z., Mansournia M.A., Yousefi B., Momen-Heravi M. (2020). An insight into the sex differences in COVID-19 patients: What are the possible causes?. Prehosp. Disaster Med..

[57] Anai M., Akaike K., Iwagoe H., Akasaka T., Higuchi T., Miyazaki A., Naito D., Tajima Y., Takahashi H., Komatsu T. (2021). Decrease in hemoglobin level predicts increased risk for severe respiratory failure in COVID-19 patients with pneumonia. Respir. Investig..

[58] Hopp M.T., Domingo-Fernández D., Gadiya Y., Detzel M.S., Graf R., Schmalohr B.F., Kodamullil A.T., Imhof D., Hofmann-Apitius M. (2021). Linking COVID-19 and Heme-Driven Pathophysiologies: A Combined Computational–Experimental Approach. Biomolecules.

[59] Cavezzi A., Troiani E., Corrao S. (2020). COVID-19: Hemoglobin, iron, and hypoxia beyond inflammation. A narrative review. Clin. Pract..

[60] Eleftheriadis T., Pissas G., Antoniadi G., Liakopoulos V., Stefanidis I. (2016). Kynurenine, by activating aryl hydrocarbon receptor, decreases erythropoietin and increases hepcidin production in HepG2 cells: A new mechanism for anemia of inflammation. Exp. Hematol..

[61] Weiss G., Schroecksnadel K., Mattle V., Winkler C., Konwalinka G., Fuchs D. (2004). Possible role of cytokine-induced tryptophan degradation in anaemia of inflammation. Eur. J. Haematol..

[62] Takahashi T., Ellingson M.K., Wong P., Israelow B., Lucas C., Klein J., Silva J., Mao T., Oh J.E., Tokuyama M. (2020). Sex differences in immune responses that underlie COVID-19 disease outcomes. Nature.

[63] Thomas T., Stefanoni D., Reisz J.A., Nemkov T., Bertolone L., Francis R.O., Hudson K.E., Zimring J.C., Hansen K.C., Hod E.A. (2020). COVID-19 infection alters kynurenine and fatty acid metabolism, correlating with IL-6 levels and renal status. JCI Insight.

[64] Webb K., Peckham H., Radziszewska A., Menon M., Oliveri P., Simpson F., Deakin C.T., Lee S., Ciurtin C., Butler G. (2019). Sex and Pubertal Differences in the Type 1 Interferon Pathway Associate With Both X Chromosome Number and Serum Sex Hormone Concentration. Front. Immunol..

[65] Drobnik W., Liebisch G., Audebert F.X., Frohlich D., Gluck T., Vogel P., Rothe G., Schmitz G. (2003). Plasma ceramide and lysophosphatidylcholine inversely correlate with mortality in sepsis patients. J. Lipid Res..

[66] Park D.W., Kwak D.S., Park Y.Y., Chang Y., Huh J.W., Lim C.M., Koh Y., Song D.K., Hong S.B. (2014). Impact of serial measurements of lysophosphatidylcholine on 28-day mortality prediction in patients admitted to the intensive care unit with severe sepsis or septic shock. J. Crit. Care.

[67] Knuplez E., Marsche G. (2020). An Updated Review of Pro- and Anti-Inflammatory Properties of Plasma Lysophosphatidylcholines in the Vascular System. Int. J. Mol. Sci..

[68] Bienvenu L.A., Noonan J., Wang X., Peter K. (2020). Higher mortality of COVID-19 in males: Sex differences in immune response and cardiovascular comorbidities. Cardiovasc. Res..

[69] Biswas M., Rahaman S., Biswas T.K., Haque Z., Ibrahim B. (2020). Association of Sex, Age, and Comorbidities with Mortality in COVID-19 Patients: A Systematic Review and Meta-Analysis. Intervirology.

